# Impact of novel lightweight disposable gastroscope and duodenoscope on endoscopist muscle activation: a comparative study

**DOI:** 10.1016/j.igie.2024.08.008

**Published:** 2024-09-03

**Authors:** Veronica Bessone, Robin Rusnak, Sven Adamsen

**Affiliations:** 1 Human Factors Engineering, Research & Development, Ambu Innovation, Augsburg, Germany; 2 Clinical Applications, Research & Development, Ambu, Ballerup, Denmark; 3 Digestive Disease Center, Copenhagen University Hospital Bispebjerg, Copenhagen, Denmark

## Abstract

**Background and Aims:**

Musculoskeletal injuries are common among endoscopists, and reducing the endoscope weight is considered to be a preventive measure. We aimed to assess the impact on muscle activation of recently developed lightweight gastroscopes and duodenoscopes, compared with standard endoscopes in a 2-part study.

**Methods:**

In the first part, 14 participants performed a protocol using a lightweight disposable and a standard reusable gastroscope in a random order. The protocol constituted of 3 equal working blocks with repetitive and standardized movements. In the second part, 15 endoscopists used a lightweight disposable and a reusable duodenoscope in an artificial model and at rest. For both protocols, the subjects wore a sleeve with embedded superficial electromyographic sensors on the left arm.

**Results:**

Wrist flexor muscle activation was significantly lower when using the lightweight single-use endoscopes during the protocol tasks (*P* < .05). The extensor muscles were also significantly less activated while using the single-use gastroscope (*P* < .05), but there was no difference when using the duodenoscopes.

**Conclusions:**

Standardized operation with lightweight endoscopes reduces the load on the left forearm muscles and favors muscle rest compared with heavier standard endoscopes. These improvements in user ergonomics may aid in preventing or delaying the development of tremor, fatigue, and injuries.

Musculoskeletal injury (MSI) is common among endoscopists.^1^, ^2^, ^3^, ^4^ Mitigation strategies for preventing MSI include optimizing the work environment and providing ergonomic training, alongside the use of ergonomically designed equipment.^3^^,^^5^^,^^6^

Like any hand tool, the endoscope weight significantly affects user comfort and the risk of developing MSI and postural tremor linked to muscle voluntary contraction,^7^ affecting performance.^8^ Therefore, reducing the endoscope’s weight has been recommended.^3^^,^^9^ Disposable endoscopes, owing to their constituent materials, are lighter than traditional reusable endoscopes. The reduced weight of single-use endoscopes is reported to diminish the muscle activation required to hold and operate the endoscope in ureteroscopy and duodenoscopy, although in case studies involving 3 or fewer subjects.^10^, ^11^, ^12^ The present study was undertaken to compare muscle activation while holding and operating gastrointestinal endoscopes of different weights in a controlled set-up in a larger group of participants.

## Methods

**Figure 1:**
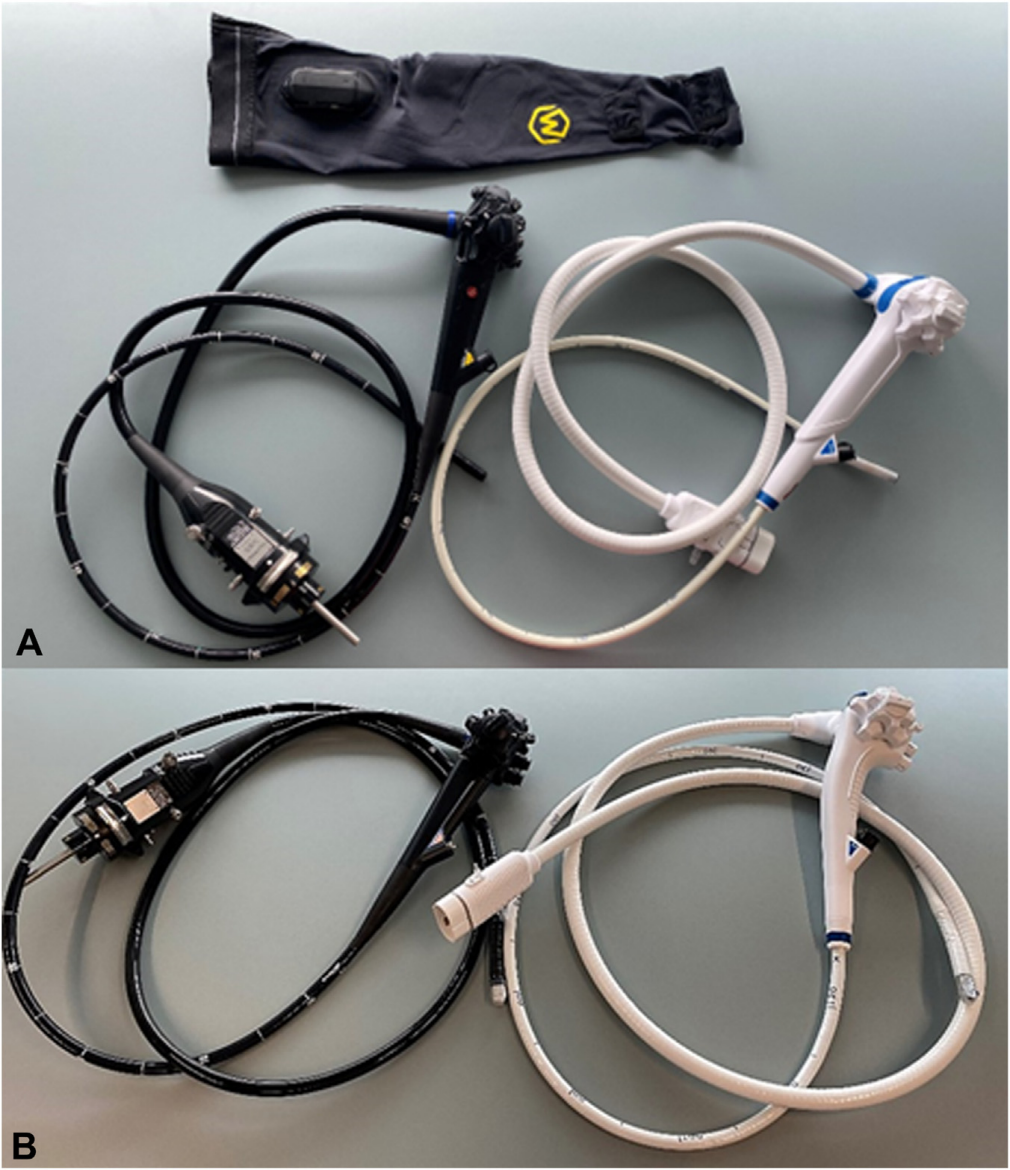
**A,** Reusable gastroscope (GIF-HQ190; Olympus) in black, disposable-use gastroscope (aScope Gastro; Ambu) in white, and ErgoSleeve (Myontec) at top. **B,** Reusable duodenoscope (TJF-Q190V; Olympus) in black and disposable-use duodenoscope (aScope Duodeno 2; Ambu) in white. The difference in handle angulation can be seen.

The study entailed 2 parts, with 14 subjects performing a protocol to compare gastroscopes (group G) and 15 comparing duodenoscopes (group D) (Table 1). The subjects participated voluntarily and were fully informed about the protocol and about the possibility of withdrawing at any point without giving a reason. Informed consent was obtained from each participant. Group G used a disposable (aScope Gastro; Ambu, Ballerup, Denmark) and a reusable (GIF-HQ190; Olympus, Tokyo, Japan) gastroscope. Group D used a disposable (aScope Duodeno 2; Ambu) and a reusable (TJF-Q190V; Olympus) duodenoscope (Fig. 1; Table 2).

**Table 1:** Characteristics of the 2 participants’ groups performing the tests with the gastroscopes (group G) and with the duodenoscopes (group D)

Group	Subjects, n	Female, n	Endoscopist, n	Median age, y	Age range, y
G	14	6	Yes: 1; No: 13	34	25-65
D	15	3	Yes: 15	46	36-65

All the subjects of group D were trained endoscopists with experience in endoscopic retrograde cholangiopancreatography.

**Table 2:** Weight characteristics of the gastroscopes and duodenoscopes used during the tests

Endoscope	Group	Disposable/reusable	Total weight, kg	Control section weight, kg
aScope Gastro	G	Disposable	0.56	0.25
GIF-HQ190	G	Reusable	1.36	0.57
aScope Duodeno 2	D	Disposable	0.64	0.32
TJF-Q190V	D	Reusable	1.36	0.54

**Figure 2:**
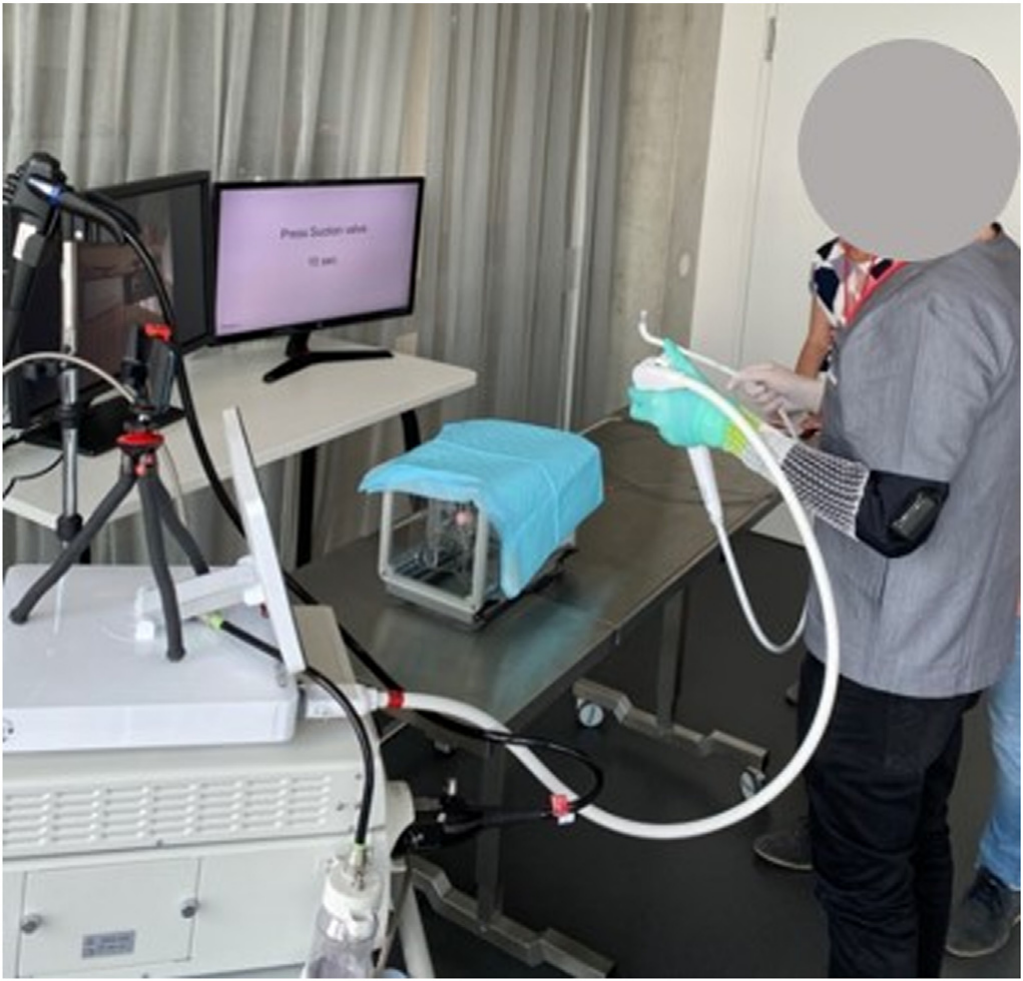
Setup overview with a participant performing with the disposable duodenoscope while wearing the sleeve with the embedded electromyographic electrodes on the left arm.

The subjects wore an ErgoSleeve (Myontec, Kuopio, Finland) on the left arm (Figs. 1 and 2) with validated embedded surface electromyography (sEMG) electrodes to detect wrist flexor and extensor muscle contractions^13^, ^14^, ^15^ (Appendix 1), connected via Bluetooth to a smartphone (Nokia, Espoo, Finland), which received and recorded a synchronized video of the test, and started and stopped the data collection. Initially the maximal voluntary contraction (MVC) was measured by performing 2 sets of minimum 5-second maximal wrist flexion, extension, abduction, and adduction.

Group G held the gastroscope in a static position for 30 seconds (rest phase), followed by 2 rotations of the up/down knob to achieve a 90° tip-up and tip-down bend, respectively, with each position held for 10 seconds. Then the insufflation and suction valves were pressed for 10 seconds each. The tasks were repeated 3 times and concluded with a 30-second rest phase.

**Figure 3:**
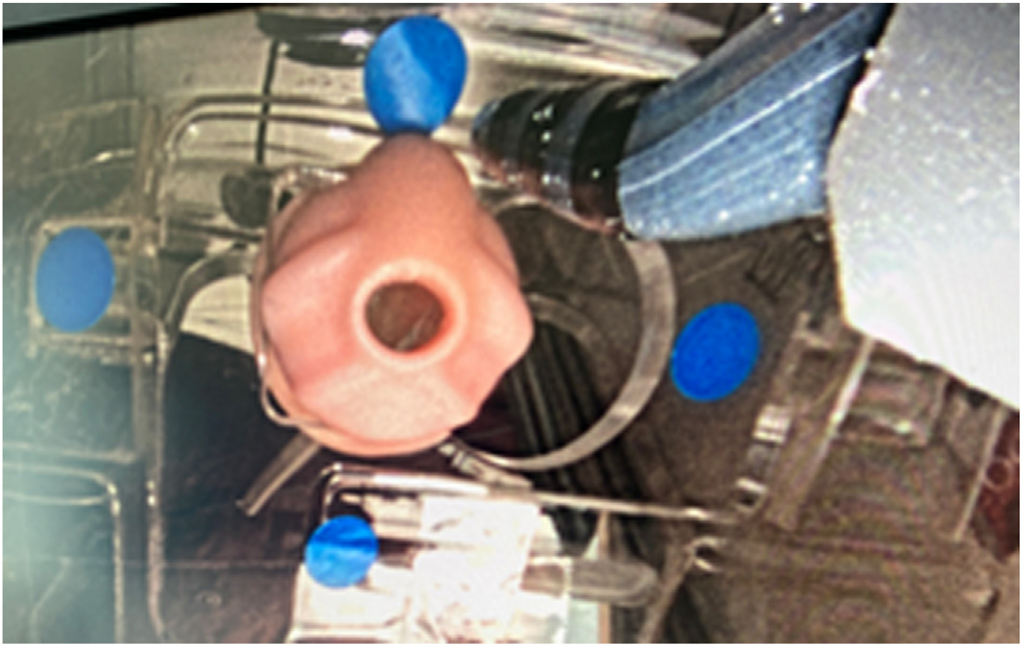
View of the artificial papilla in the Boškoski-Costamagna endoscopic retrograde cholangiopancreatography (ERCP) trainer and position of the 4 blue dots to be touched with the sphincterotome.

Group D started with a 30-second rest phase. Then the endoscope was inserted in the Boškoski-Costamagna endoscopic retrograde cholangiopancreatography (ERCP) trainer (Cook Medical, Bloomington, Ind, USA) and advanced to the papilla, which was cannulated with a Fusion Omni sphincterotome (Cook Medical) (Fig. 2). To increase task complexity, the subject touched 4 blue adhesive dots positioned round the papilla with the sphincterotome’s tip (Fig. 3).

To automate both protocols, the instructions and timing of the exercises were displayed on a monitor in front of the subjects (Fig. 2). The order of using the disposable and reusable endoscopes was randomized. To avoid bias, the participants were not instructed on how to hold the endoscopes. After the tests, the sEMG data were processed with the software Ergolink (Myontec) to calculate the muscle load (%MVC; percentage of the normalization of the sEMG signals on MVC) and the microbreaks (%t; percentage relative to the duration of the block of periods [lasting much less than 1 s] when %MVC is <0.5 and considered as “recovery time”). Data were reported as mean ± standard deviation and analyzed with the use of paired *t* tests, with statistical significance set at *P* < .05 and calculated with the use of Microsoft Excel (Microsoft Corporation, Redmond, Wash, USA).

## Results

Flexors’ and extensors’ %MVCs were significantly higher and microbreaks were significantly more frequent when using the reusable standard gastroscope during both the rest phase and the overall protocol (Table 3).

**Table 3:** Muscle loads and microbreaks with the disposable and the reusable gastroscopes

Phase	Variable	Disposable gastroscope	Reusable gastroscope	% change compared with reusable	*P* value
Rest phase	%MVC_ext_	3 ± 2	4 ± 2	−28 ± 21	<.05
	%MVC_flex_	2 ± 1	3 ± 1	−27 ± 22	<.001
	%t_ext_	95 ± 12	79 ± 29	–	<.05
	%t_flex_	89 ± 12	75 ± 24	–	<.01
Overall protocol	%MVC_ext_	5 ± 2	6 ± 2	−16 ± 11	<.005
	%MVC_flex_	6 ± 3	7 ± 3	−17 ± 17	<.001
	%t_ext_	67 ± 5	52 ± 23	–	<.005
	%t_flex_	44 ± 18	35 ± 19	–	<.001

Values are presented as mean ± standard deviation. Muscle load of the wrist flexors (%MVC_flex_) and extensors (%MVC_ext_), muscle load change in percentage between the disposable and the reusable gastroscopes (negative value indicates a reduction, positive value an increase), microbreaks for the flexors (%t_flex_) and extensors (%t_ext_) at rest and during the overall protocol while using the gastroscopes.

For the duodenoscope, flexor %MVC and %t were lower and higher, respectively, with the disposable duodenoscope during the tasks and the rest phase (Table 4). No difference was found for extensors. The artificial model task lasted 102 ± 38 seconds with the disposable duodenoscope and 98 ± 32 seconds with the reusable duodenoscope (*P* > .05).

**Table 4:** Muscle loads and microbreaks with the disposable and the reusable duodenoscopes

Phase	Variable	Disposable duodenoscope	Reusable duodenoscope	% change compared with reusable	*P* value
Rest phase	%MVC_ext_	3 ± 2	4 ± 3	1 ± 69	>.05
	%MVC_flex_	2 ± 1	3 ± 1	−28 ± 27	<.01
	%t_ext_	86 ± 23	73 ± 37	–	>.05
	%t_flex_	83 ± 33	54 ± 44	–	<.01
Artificial model	%MVC_ext_	21 ± 14	21 ± 13	−2 ± 20	>.05
	%MVC_flex_	6 ± 3	8 ± 4	−21 ± 15	<.001
	%t_ext_	1 ± 2	1 ± 2	–	>.05
	%t_flex_	11 ± 10	5 ± 7	–	<.01

Values are presented as mean ± standard deviation. Muscle load of the wrist flexors (%MVC_flex_) and extensors (%MVC_ext_), muscle load change in percentage between the disposable and the reusable duodenoscopes (negative value indicates a reduction, positive value an increase), and microbreaks for the flexors (%t_flex_) and extensors (%t_ext_) during the rest phase and the artificial model task while using the duodenoscopes.

## Discussion

The main findings of the study are that (1) while holding and maneuvering the lightweight disposable gastroscope, the extensor muscle load and microbreaks are significantly reduced and increased, respectively, and (2) while holding and maneuvering the lightweight disposable gastroscope and duodenoscope, the flexor muscle load and microbreaks are significantly reduced and increased, respectively.

The findings align with a previous study reporting a reduced upper arm %MVC when performing ERCP with a disposable compared with a reusable duodenoscope in a desktop model, explained by the lighter weight of the disposable endoscope which leads to a reduced static and dynamic load,^12^ using a standard EMG system. Even though the endoscope weight is partially distributed along the insertion tube and connection cord, the total weight of standard endoscopes is twice that of the disposables, explaining the significantly lower %MVC for the latter. In another study it was observed that heavier hand tools led to earlier detection of postural tremor, occurring after 9 minutes for tools weighing 0.8 kg versus 12 minutes for tools weighing 0.4 kg.^8^ Given that most diagnostic gastroscopies last up to 10 minutes,^1^ we presumed that tremor might occur more frequently when heavier endoscopes are used. Especially in long procedures, such as ERCP,^1^^,^^3^ reducing the endoscope weight could be a significant improvement to optimize ergonomics and reduce fatigue and musculoskeletal strain,^12^^,^^15^ leading to a possible reduction of the procedure time, as demonstrated in ureteroscopy.^16^

**Figure 4:**
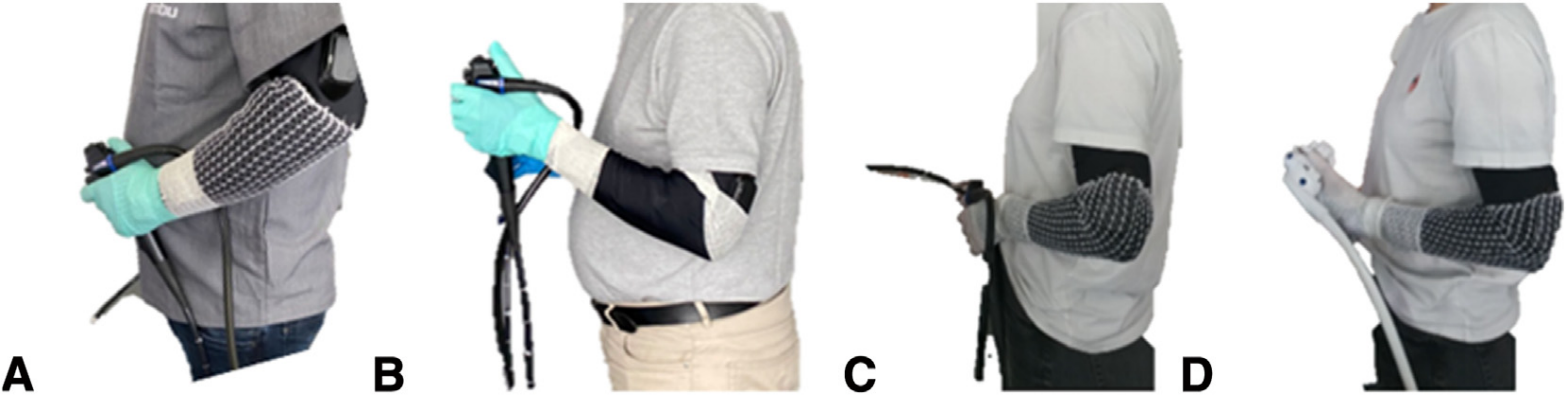
Three subjects (**A,B,** and **C**) holding the reusable endoscope in the rest position. Same subject in **C** and **D** holding the two duodenoscopes differently in the rest position.

Extensors’ %MVC was higher than flexors’ for equal tasks owing to the lower force-generating capacity of extensors^17^ and the greater contribution to wrist stability during complex motor tasks.^18^^,^^19^ Extensors’ %MVC and %t did not differ between the 2 duodenoscopes. This could be explained by the influence of different intra- and intersubject left arm postures while holding the duodenoscopes, influencing the umbilical cord placement and the elbow angles^12^^,^^20^^,^^21^ (Fig. 4), as well as by the different control sections’ design.

The rate of microbreaks was significantly longer while using the lightweight endoscopes, meaning that the muscles are actively recovering more, which delays the development of fatigue. In the rest phase, when the endoscope mechanics does not affect muscle activation, %t and %MVC were influenced exclusively by the endoscope weight.

The study was not performed in a clinical setting, limiting its reliability. The many different movements in clinical procedures depend on the various indications, making standardization of movements virtually impossible. For this reason, standardized movements limiting intra- and intersubject variability were used in this study. Another limitation was the relatively low number of subjects, although similar to^22^, ^23^, ^24^ or higher than^10^, ^11^, ^12^^,^^25^ previous publications on muscle physiology in humans in endoscopy. Finally, the novel handle design of the lightweight duodenoscope may have influenced the results.

In conclusion, holding and operating lightweight disposable endoscopes significantly decreases the strain on the left wrist muscles compared with heavier reusable endoscopes. This improvement in user ergonomics may help mitigate or postpone the onset of tremor, fatigue, and MSI. Further research should focus on the effects of using lightweight endoscopes by collecting biomechanical and physiologic data with wearable sensors in clinical procedures.

## Disclosure

The following authors disclosed financial relationships: V. Bessone and R. Rusnak: employed by Ambu Innovation GmbH. S. Adamsen: medical advisor for Ambu A/S.
