# Supplementary material for: Impact of novel lightweight disposable gastroscope and duodenoscope on endoscopist muscle activation: a comparative study
Source: IGIE. 2024 Sep 3;4(1):6–10. doi: 10.1016/j.igie.2024.08.008 (PMC12850842; doi:10.1016/j.igie.2024.08.008)
Supplement: Appendix 1 [file mmc1.docx]

**Appendix A**

Electromyography (EMG) is a common technique utilized to detect the muscle activation of subjects for diagnostics, for research and for performance analysis. Standard EMG sensors are connected to electrodes placed on the skin through cables [13] and can detect the muscle activation beneath one point of skin contact [22]. To perform EMG analysis a proper skin preparation with shaving and scrubbing is necessary before precisely placing the electrodes on the muscles. Additionally, to ensure that the electrodes and sensors are not detaching from the skin, these must be secured with medical tape or grid. This preparation usually takes considerable time and can be performed only by trained physiologists or biomechanists in order to locate the correct muscles to test.

Textile electrodes embedded in cloths (shirt, short or sleeve) as the one utilized in the study, do not require such meticulous sensor placement and are wireless, allowing a fast subject preparation without movement constraints, and the freedom of using the systems in elite training and rehabilitation environments as well as in daily and working life activities for ergonomic purposes [14,15]. Disadvantage of these systems is that the electrodes are covering a wider area, and therefore, the activation of one single muscle cannot be detected, but only the one of a group of muscles (such as for instance the wrist flexors and extensors).

The ErgoSleeve (Myontec Ltd., Kuopio, Finland), utilized in the present study, is a sleeve with embedded superficial electromyography (sEMG) electrodes to detect wrist flexor and extensor muscle contractions and inertial motion units (IMUs) to assess the wrist angle. The sEMG system records with a sampling rate of 1000 hertz (Hz), while the IMUs at 30 Hz. ErgoSleeve is a validated system and used in previous published studies by several groups [13-15,24]. ErgoSleeve is available in different sizes (small, medium, and large) that can be used depending on the anthropometrics of the tested subject.

The ErgoSleeve is connected via Bluetooth to a smartphone (Nokia, Espoo, Finland), which works as a receiver, to record a synchronized video of the test protocol, to start and stop, and to download the data into the Ergolink software (Myontec Ltd., Kuopio, Finland). The software is then utilized to process the data.

**Disclosures:**

The following authors disclosed financial relationships: V. Bessone and R. Rusnak: Employed by Ambu Innovation GmbH. S. Adamsen: Medical advisor for Ambu A/S.

Preliminary results of the gastroscope study were presented at the United European Gastroenterology Week 2023.

**Acronyms used in the manuscript:**

MSI, musculoskeletal injuries; group G, group of participants testing the gastroscopes; group D, group of participants testing the duodenoscopes; sEMG, superficial electromyography; MVC, maximal voluntary contraction; %MVC, muscle load; %MVC_flex_, flexor muscle load; %MVC_ext_, extensor muscle load; %t, microbreak; %t_flex_, flexor muscle microbreak; %t_ext_, extensor muscle microbreak

V.Bessone, R.Rusnak and S.Adamsen contributed equally to this article.

Current affiliations: Human Factors Engineering, Research & Development, Ambu Innovation GmbH, Augsburg, Germany (1), Clinical Applications, Research & Development, Ambu A/S, Ballerup, Denmark (2), Digestive Disease Center, Copenhagen University Hospital Bispebjerg, Copenhagen, Denmark (3).

Reprint requests: Veronica Bessone, PhD, Ambu Innovation GmbH, Karl-Drais-Strasse 4B, DE-86159, Augsburg, Germany. E-mail: vebe@ambu.com

**References**

1. Bessone V, Adamsen S. Gastrointestinal endoscopy and work-related injuries: an international survey. Endosc Int Open 2022;10:E562-9.
2. El Bacha H, Mariam K, Nadia B, Errabih I. Prevalence of musculoskeletal disorders among digestive endoscopy physicians. Endoscopy 2023;55:S85.
3. Bessone V, Roppenecker DB, Adamsen S. Work-Related Musculoskeletal Injury Rates, Risk Factors, and Ergonomics in Different Endoscopic Specialties: A Review. Healthcare. 2024;12:885.
4. Singh AD, Aggarwal N, Mohan BP, et al. Prevalence of endoscopy-related injuries and their impact on clinical practice: a systematic review and meta-analysis. Endoscopy 2024 Apr 25. doi: 10.1055/a-2270-4174. Online ahead of print. Accessed July 11^th^, 2024
5. Lipowska AM, Shergill AK. Ergonomics of endoscopy. Gastrointest Endosc Clin N Am 2021;31:655-69.
6. Gimpaya N, Tran WT, Grover SC. Ergonomic wellness for the trainee in gastrointestinal endoscopy. Curr Opin Gastroenterol 2024 Apr 17. doi: 10.1097/MOG.0000000000001034. Online ahead of print. Accessed July 11^th^, 2024
7. Sato I, Yamamoto S, Kakimoto M, et al. Basic characteristics between mechanomyogram and muscle force during twitch and tetanic contractions in rat skeletal muscles. J Electromyogr Kinesiol 2022;62:102627.
8. Kim JY, Song HY, Yun MH, Yun MW. Effect of tool weight and hand posture on the postural tremor of the upper extremity. J Ergon Soc Korea 1996;15:177-84.
9. Khanicheh A, Shergill AK. Endoscope design for the future. Tech Gastrointest Endosc 2019;21:167-73.
10. Ludwig WW, Lee G, Ziemba JB, Ko JS, Matlaga BR. Evaluating the Ergonomics of Flexible Ureteroscopy. J Endourol. 2017;31(10):1062-1066.
11. Wright HC, Gheordunescu G, O'Laughlin K, et al. Ergonomics in the OR: An electromyographic evaluation of common muscle groups used during simulated flexible ureteroscopy - a pilot study. Urology 2022;170:66-72.
12. Cennamo V, Botter A, Landi S, et al. Can single-use versus standard duodenoscope improve ergonomics in ERCP? A comparative, simulation-based pilot study. Endosc Int Open 2024;12:E419-27.
13. Tikkanen O, Hu M, Vilavuo T, et al. Ventilatory threshold during incremental running can be estimated using EMG shorts. Physiol Meas 2012;33(4):603-14.
14. Colyer SL, McGuigan PM. Textile electrodes embedded in clothing: A practical alternative to traditional surface electromyography when assessing muscle excitation during functional movements. J Sports Sci Med 2018;17:101-9.
15. Hermann A., Senner V. EMG-pants in sports: Concept validation of textile-integrated EMG measurements. In: Proceedings of the 8th International Conference on Sport Sciences Research and Technology Support (icSPORTS 2020), pp 197-204. ISBN: 978-989-758-481-7
16. Salvadó JA, Cabello JM, Moreno S, et al. Endoscopic treatment of lower pole stones: is a disposable ureteroscope preferable? Results of a prospective case-control study. Cent European J Urol 2019;72:280-84.
17. Forman GN, Forman DA, Avila-Mireles EJ, et al. Investigating the muscular and kinematic responses to sudden wrist perturbations during a dynamic tracking task. Sci Rep 2020; 5;10(1):4161
18. Holmes MWR, Tat J, Keir PJ. Neuromechanical control of the forearm muscles during gripping with sudden flexion and extension wrist perturbations. Comput Methods Biomech Biomed Eng 2015;18:1826–34.
19. Singh M, Karpovich PV. Strength of forearm flexors and extensors in men and women. J Appl Physiol 1968;25:177–80.
20. Roman-Liu D, Bartuzi P. The influence of wrist posture on the time and frequency EMG signal measures of forearm muscles. Gait Posture 2013;37:340-44.
21. Harris-Adamson C, Shergill AK. Endoscopist injury: shifting our focus to interventions. Gastrointest Endosc 2021;94:260-2.
22. Gilbert CR, Thiboutot J, Mallow C, et al. Assessment of ergonomic strain and positioning during bronchoscopic procedures: A feasibility study. J Bronchology Interv Pulmonol 2020;27:58-67.
23. Kim E, Sun A, Rodriguez-Alvarez JS, et al. Gender differences in ergonomics during simulated ureteroscopy. Am J Surg 2024 Feb 22. Online ahead of print. https://doi.org/10.1016/j.amjsurg.2024.02.034. Accessed July 11th, 2024
24. Durič T, Cibulkova I, Hajer J. Prevention of musculoskeletal injuries in gastrointestinal endoscopists. Gastroenterology Insights 2024;15:285-302.
25. Shergill AK, Asundi KR, Barr A, et al. Pinch force and forearm-muscle load during routine colonoscopy: a pilot study. Gastrointest Endosc. 2009;69:142-6.
